# Cholesterol Disturbances and the Role of Proper Nutrition in CKD Patients

**DOI:** 10.3390/nu11112820

**Published:** 2019-11-18

**Authors:** Anna Gluba-Brzozka, Beata Franczyk, Jacek Rysz

**Affiliations:** Department of Nephrology, Hypertension and Family Medicine, Medical University of Lodz, 90-549 Lodz, Poland; bfranczyk-skora@wp.pl (B.F.); jacek.rysz@umed.lodz.pl (J.R.)

**Keywords:** lipid profile, chronic kidney disease, diet, cardiovascular risk

## Abstract

Chronic kidney disease (CKD) is a widespread disease with increasing prevalence in the modern society. Lipid disturbances are common in this group of patients. In most patients with CKD atherogenic dyslipidemia is observed. Dyslipidemia in patients with renal diseases increases the risk of cardiovascular diseases and it accelerates the progression of chronic kidney disease to its end stage. The amelioration of dyslipidemia and the lowering of oxidative stress, inflammatory processes, insulin sensitivity and remnant lipoproteins levels may lead to the reduction in cardiovascular burden. Nutritional interventions can strengthen the beneficial effect of treatment and they play an important role in the preservation of overall well-being of the patients with CKD since the aim of appropriate diet is to reduce the risk of cardiovascular events, prevent malnutrition, and hamper the progression of kidney disease. The management of dyslipidemia, regardless of the presence of chronic kidney disease, should be initiated by the introduction of therapeutic lifestyle changes. The introduction of diet change was shown to exert beneficial effect on the lipid level lowering that reaches beyond pharmacological therapy. Currently available evidence give the impression that data on dietary interventions in CKD patients is not sufficient to make any clinical practice guidelines and is of low quality.

## 1. Introduction

Chronic kidney disease (CKD) is a widespread disease with increasing prevalence in modern societies [1]. Lipid disturbances are common in this group of patients. In most patients with CKD atherogenic dyslipidemia is observed [2]. Dyslipidemia present in patients with renal diseases not only increase the risk of cardiovascular diseases but also it accelerates the progression of chronic kidney disease to its end stage [1]. The amelioration of dyslipidemia and lowering of oxidative stress, inflammatory processes, insulin sensitivity, and remnant lipoproteins levels may lead to the reduction in cardiovascular burden [1]. Hypercholesterolemia, hypertriglyceridemia, and increased levels of low-density lipoprotein-cholesterol are thought to be crucial factors for cardiovascular disease (CVD) risk in CKD patients [3]. Nutritional interventions can strengthen beneficial effect of treatment and they play an important role in the preservation of the overall well-being of the patients with chronic renal failure since the aim of appropriate diet is to reduce cardiovascular events risk, prevent malnutrition, and hamper the progression of kidney disease [3,4,5,6].

## 2. Cholesterol Disturbances in CKD

**Figure 1:**
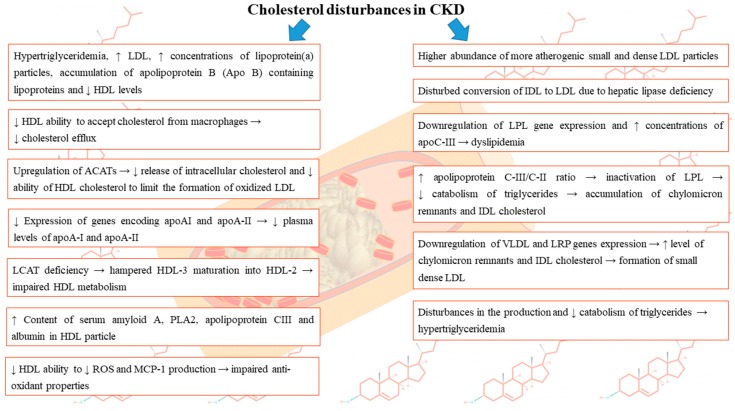
Summary of disturbances in lipoproteins levels occurring in the course of CKD.

Numerous studies indicated a relationship between renal dysfunction and disturbances in lipoprotein metabolism which results in dyslipidemia and subsequent accumulation of atherogenic particles [7]. In the course of chronic kidney disease the following disturbances are frequently observed: elevated levels of VLDL, IDL, triglycerides, normal to increased levels of LDL, but at the same time elevated concentrations of oxidized LDL, HDL deficiency and dysfunction, diminished levels of apolipoprotein A-1, the accumulation of apolipoprotein B (Apo B)-containing lipoproteins, as well as higher apolipoprotein C-III/C-II ratio [7]. Abnormalities concerning lipoproteins vary depending on the renal impairment degree, primary disease etiology, the presence of nephrotic syndrome (NS) and in case of patients requiring renal replacement therapy also the method of renal replacement therapy—hemodialysis (HD) or peritoneal dialysis (PD) [8]. The altered lipoprotein levels can stimulate the progression of kidney function impairment [9,10]. The disturbances in lipoproteins levels occurring in the course of CKD are summarized in Figure 1.

### 2.1. Cholesterol Disturbances in CKD Stage 2–3a, 3b, and 4

Some studies indicated enhanced triglycerides concentrations in patients with impaired renal function despite serum creatinine levels within normal limits [8,11,12]. In these patients, postprandial lipemia (abnormal raise in serum triglyceride levels after a fat meal) is also observed, which can be associated with disturbed clearance of chylomicron remnants [13,14]. The occurrence of hypertriglyceridemia, which is one of the most common quantitative lipid abnormalities in patients with CKD, especially in its early stages, is associated with disturbances in triglycerides production as well as their decreased catabolism [8,15,16,17]. The diminished catabolic rate has been suggested to be associated with reduced lipoprotein lipase (LPL) activity resulting in hampered (LPL)-mediated hydrolysis of triglycerides in VLD and chylomicrons [18], thus leading to the accumulation of chylomicron remnants and IDL cholesterol [16,19]. The increase in chylomicron remnants is related to the downregulation of the expression of a gene encoding very low density lipoprotein (VLDL) and low density lipoprotein (LDL) receptor-related protein (LRP) [20,21]. Animal studies confirmed the existence of inverse correlation between plasma triglycerides and LPL activity [18]. Some studies confirmed that in CKD, adipose tissue LPL activity is diminished [22,23]. Also, PTH-induced insulin resistance and the presence of surplus lipase inhibitors in uremic plasma have been attributed to lower LPL activity in CKD [8,18]. Vaziri et al. [18] demonstrated that CKD stimulate significant downregulation of LPL expression, which is related to the presence of markedly elevated levels of plasma of Apo C-III and pre-beta-HDL. These two factors are potent inhibitors of LPL. In uremic patient a decrease in apolipoprotein C-II (activator of LPL)/C-III (inhibitor of LPL) ratio is frequently observed [24,25]. Also, the presence of secondary hyperparathyroidism may be involved in the compromised catabolism of triglyceride-rich lipoproteins, thus leading in consequence to elevated plasma triglyceride concentrations [23,26]. Dyslipidemia in renal disease is aggravated by enhanced hepatic production of triglyceride-rich lipoproteins [27]. Hepatic VLDL production can be stimulated by the presence of insulin resistance in CKD patients [11,12]. It has been suggested that excessive insulin resistance-associated production of VLDL is another factor contributing to hypertriglyceridemia in patients with CKD [8]. In the early stages of chronic kidney disease, high levels of LDL-cholesterol are observed. The results of National Observatory of Atherosclerosis in Nephrology (the NEFRONA Study) indicate a progressive decrease in total cholesterol, LDL-cholesterol, HDL-cholesterol, and non-HDL-cholesterol levels with increasing stage of renal disease [28]. Despite the fact that the levels of serum LDL cholesterol can be within normal range in CKD patients, the concentration of atherogenic sdLDL increases along with the progression of kidney function deterioration [16].

Hepatic lipase (HL) deficiency which is observed in chronic renal failure results in defective IDL to LDL transformation, increase in serum IDL, TG-enrichment of LDL, and hypertriglyceridemia [20,29]. Studies on rats revealed that in chronic kidney disease the mRNA of hepatic lipase is downregulated and therefore HL production, activity, and release are disturbed. It has been suggested that the mechanism of this phenomenon is related to secondary hyperparathyroidism since the prevention of excess PTH by PTX or the treatment with verapamil (which blocks the effects of PTH) corrected compromised hepatic lipase metabolism [29]. Moreover, the clearance of VLDL is impaired in chronic renal failure because of significant downregulation of VLDL receptor gene expression and protein abundance which leads in consequence to increased plasma levels of VLDL and triglyceride [20,30,31].

In comparison to individuals with preserved kidney function, in patients with CKD decreased HDL levels are observed [32]. Several processes are implicated in the reduction of serum levels of HDL. First, impaired renal function is associated with decreased plasma levels of main HDL components—apoA-I and apoA-II, as a result of diminished expression of genes encoding apoAI and apoA-II [20]. In vitro studies demonstrated that downregulation of apoAI expression is related to the presence of uremic toxins and mediated by mRNA instability [33]. The level of functional apolipoprotein A1 can be also decreased by modifications by reactive oxygen species (oxidation), elevated urea level (carbamylation), and systemic inflammation (myeloperoxidase modification) [34]. Moreover, in patients with chronic kidney disease, the activity of lecithin–cholesterol acyltransferase (LCAT), which is responsible for the esterification of free cholesterol in HDL, is compromised [32]. Guarnieri et al. [35] suggested that LCAT synthesis in the liver might be reduced in chronic uremia. This decrease was shown to be a consequence of reduced transcription of the LCAT gene in the liver [36]. This observation was confirmed in animal model which revealed marked decline of hepatic tissue LCAT mRNA abundance [36]. The reduction in hepatic LCAT mRNA was shown to be accompanied by a significant diminishing of plasma LCAT activity. It seems that it is the inflammatory state that contributed to the downregulation of hepatic LCAT production in CRF [36]. According to studies, the impairment of HDL metabolism in CKD is due to lecithin-cholesterol acyl-transferase (LCAT) deficiency that leads to hampered HDL-3 maturation into HDL-2 [36,37]. Furthermore, cholesterol ester transfer protein (CETP) activity is increased which results in enhanced transfer of cholesterol esters from HDL to triglyceride-rich lipoproteins. In normal conditions, the rate of CETP-mediated cholesteryl ester transfer depends on the rate of HDL and LDL catabolism [38,39]. However, in a state when elevated concentration of VLDLs is observed, HDL cholesteryl esters are preferentially transferred by CETP to larger VLDL particles [38,40].

Apart from diminished HDL levels, in CKD also decreased activity of HDL-associated enzymes, including paraoxonase is observed [32]. 

### 2.2. Cholesterol Disturbances in ESRD (5ND)

According to studies, ESRD-related dyslipidemia is characterized by hypertriglyceridemia, elevated plasma concentration of lipoprotein remnants and very low density lipoprotein (VLDL), the accumulation of oxidized lipids and lipoproteins, decreased levels of plasma HDL cholesterol, as well as impaired HDL maturation and function [20,41]. Moreover, in ESRD patients, LDL cholesterol is not usually elevated, however, these particles tend to be smaller, denser, and more atherogenic [32]. They contain abnormal levels of residual triglycerides [20,42]. Delayed catabolism of triglycerides in pre-dialysis patients, is a major mechanism responsible for elevated concentration of triglyceride-rich lipoproteins [8,27]. In ESRD patients, the level of oxidized LDL and IDL particles is increased [32]. Prolonged time of lipoproteins residence in circulation because of considerably modified lipid subfraction turnover enables their post-ribosomal modification which includes glycation, oxidation, and carbamylation. Such modifications result in reduced affinity of these particles to classic LDL receptors, however, the number of scavenger receptors which take up altered lipoproteins is increased leading to the accumulation of cholesterol, the formation of foam cells in the vascular walls, and finally to the development of atherosclerotic plaques [32].

Decreased levels of HDL in ESRD may be associated with significantly reduced plasma concentration of ApoA-I, since it is the crucial protein constituent of HDL [20]. Also hypoalbuminemia, which is often present in the ESRD as a result of inflammation, malnutrition, etc., possibly contribute to lower serum HDL levels due to the fact that HDL receives a considerable amount of its cholesterol content from albumin. Albumins act as a carrier of free cholesterol from the peripheral tissues to HDL-3 [20,43]. 

In ESRD patients, significantly reduced plasma LCAT activity and concentration are observed [37,44]. In end-stage renal disease (ESRD) patients, non-enzymatic glycation of apoA-I results in a diminished activation of LCAT leading in consequence to higher apoA-I clearance from circulation and in hampered anti-inflammatory and antioxidant properties of HDL [45]. The deficiency of LCAT not only contribute to the decrease of HDL level, impaired HDL maturation, and the increase in serum levels of pre-beta HDL particles, but also it can hasten the degradation of HDL [20]. The accelerated degradation of HDL is associated with preferential binding to endocytic receptor in the liver leading to immature HDL particles internalization and degradation in ESRD population [20]. Among additional factors which decrease lipoprotein lipase activity in ESRD there are the following: low levels of mature HDL particle (which is ApoE and ApoC donor) but high levels of pre-beta HDL (which act in an opposite way), diminished ApoC-II/ApoC-III ratio, lower physical activity, disturbed thyroxin (T4) to-tri-iodothyronin (T3) conversion, as well as insulin resistance [20]. HDL from patients with end-stage CKD has been demonstrated to be pro-oxidant because of the fact that it undergoes oxidative modification [37].

According to studies, ESRD-related changes in the pattern of plasma lipoproteins can be markedly altered by dialysis modality [20]. For example, serum cholesterol and LDL cholesterol levels are frequently within or below the normal limits in ESRD patients undergoing hemodialysis, while in patients on peritoneal dialysis they are increased.

### 2.3. Cholesterol Disturbances in Dialyzed Patients

#### 2.3.1. Hemodialysis

The HD procedure involves factors that may affect lipoprotein metabolism [46]. In dialysis patients, dyslipidemia rather than hyperlipidemia is frequently observed [42]. According to studies, in up to 70% of HD patients, hyperlipidemia, mainly in the form of moderate elevation of plasma triglycerides is present [46]. Slightly lower concentrations of triglyceride-rich lipoproteins in HD patients in comparison to patients before dialysis may suggest the attenuation of dyslipidemia [47]. Hemodialysis (HD) patients usually display increased TG, reduced serum HDL cholesterol, and increased levels of lipoprotein-a (Lp-a). The pathogenesis of ESRD-induced hypertriglyceridemia and impaired VLDL and chylomicron metabolism is associated with severe lipoprotein lipase deficiency resulting from the repetitive use of heparin which enables the detachment of endothelium-bound enzyme and its consequent removal by LDL receptor-related protein (LRP) in the liver and downregulation of lipoprotein lipase expression by elevated levels of parathyroid hormone [20,41]. Some articles suggest that the use of low-molecular weight heparins as an anticoagulation procedure results in a moderate reduction of triglyceride levels in comparison with unfractionated heparin [46]. Ottosson et al. revealed that choice of dialysis membrane does not influence the dyslipidemia [48].

Total and LDL cholesterol levels as well as non-HDL cholesterol usually remain within normal limits [42,49]. In patients with ESRD undergoing hemodialysis, reduced level of apoAI are observed which is associated with its enhanced catabolism. Moreover, in these patient the higher levels of anti-apoAI autoantibodies has been observed which results in diminished levels of ApoAI and the dysfunction of these proteins [33]. Attman et al. [47] have suggested that in dialysis patients moderate elevations of apoB and apoE, and a significant increase of apoC-III concentrations are observed. In HD patients the levels of ApoA-IV are increased. The elevation of apoC-III and VLDL-cholesterol and lower levels of HDL cholesterol are seen even in patients without hyperlipidemia [46,47,50]. Attman et al. [46,47] also demonstrated the increase of apoC-III in apoB-containing lipoproteins and in the levels of triglyceride-rich apoB-containing lipoproteins, which resulted in a significant rise in apoB-containing lipoproteins in IDL9. 

The results of study carried out by Lee et al. revealed no significant differences in the reactivity of lipoprotein lipase between VLDL and IDL from HD and from pre-dialysis patients [51]. 

Some studies indicated lack of significant differences in plasma CETP concentration between hemodialysis patients and normal subjects [20,52,53]. Chronic kidney disease not only stimulates the reduction of HDL concentration, but also alters the composition of this lipoprotein [54]. Because of the presence of enhanced systemic inflammation and oxidative stress, the reduction of anti-oxidant, anti-inflammatory functions of HDL, or even the conversion of HDL into a pro-oxidant/pro-inflammatory particle have been observed [20,55]. In hemodialysis patients significant reduction of plasma levels of paraoxonase and glutathione peroxidase accompanied by severe loss of HDL anti-oxidant capacity are observed [37]. Yamamoto et al. [56] demonstrated elevated content of serum amyloid A, PLA2, apolipoprotein CIII, and albumin in HDL particle coming from dialyzed patients. Moreover, such HDL particle ability to accept cholesterol from macrophages was reduced which, in consequence, resulted in impaired cholesterol efflux. Ribeiro et al. [57] demonstrated substantial accumulation of oxLDL, especially in HD patients. It seems that the main features of renal dyslipidemia remain principally unchanged during HD, however, the dyslipidemia can be moderately attenuated during long-term HD [46].

#### 2.3.2. Peritoneal Dialysis (PD)

In the available literature, there are fewer studies concerning dyslipidemia in PD patients than in HD patients. Numerous studies indicate that hyperlipidemia is more prevalent in PD than in HD patients [46,47,58,59]. Moreover, in this group of patients, also the increase of plasma cholesterol and LDL-cholesterol levels are noted [46,47,58,59]. While in patients without heavy proteinuria LDL levels are usually normal or slightly reduced, in patients on peritoneal dialysis, LDL are frequently increased, as a result of protein loss leading to considerable upregulation of 3-hydroxy-3-methylglutaryl-CoA (HMG-CoA) reductase gene expression and decreased expression of hepatic LDL receptors [15]. When apolipoprotein profile of PD patients is concerned, a proportionately greater elevation of apoB, apoC-III, and apoE levels is reported in comparison to HD patients [46,47,58]. Not only triglyceride-rich apoB-containing lipoproteins levels are increased but also cholesterol-rich apoB-containing lipoproteins, which means that also IDL and LDL levels are higher [46,47,58]. Similarly to HD patients and patients with less advanced renal failure, also in peritoneal dialysis the decrease of apoA-containing lipoproteins in HDL is visible [46,47,58]. The aforementioned disturbances observed in patients undergoing peritoneal dialysis are associated with some specific features of PD treatment that influence the lipoprotein metabolism. Marked absorption of glucose from the dialysis fluid enhances lipoprotein synthesis and in consequence it leads to higher plasma lipid concentrations [59]. Bredie et al. [60] demonstrated that the use of icodextrin-containing dialysis solutions instead of glucose for the overnight dwell resulted in a moderate decrease of plasma cholesterol. Protein clearance in the course of peritoneal dialysis involves the leakage of albumins, but also apolipoproteins and HDL which may trigger mechanisms similar to those present in nephrotic syndrome, mainly the rise in cholesterol-rich lipoproteins [46,47,58]. 

PD patients in comparison to HD patients have more pronounced increase in atherogenic lipoproteins [46].

### 2.4. Cholesterol Disturbances in Transplant Patients

According to studies, the impairment of lipid metabolism is frequent before renal transplantation [61]. After transplantation and renal function recovery, various metabolic derangements of chronic renal failure reverse but lipid disorders appear to progress in a large number of patients [62]. Lipid disturbances usually show a different profile because of various effects of immunosuppressive drugs [63,64], including calcineurin inhibitors (cyclosporine and tacrolimus), corticosteroids, antiproliferative drugs (azathioprine), mammalian target of rapamycin inhibitors (sirolimus and everolimus) [62]. Among renal transplant recipients, elevated total cholesterol, LDL cholesterol, and triglyceride concentrations as well as and decreased HDL cholesterol levels are most frequent [65]. However, the increase in HDL cholesterol is seen in patients treated with corticosteroids, including prednisone and deflazacort. Other study indicated that significant elevation in HDL following transplantation occurs in time similar to half-life for HDL biosynthesis, thus authors suggested that kidney transplantation may have a rapid effect on HDL metabolism [66]. They also observed that after this increase, HDL levels were quite stable over a three-year follow-up time, so they hypothesized that kidney transplantation exerts not only rapid but also long-lasting effects on HDL concentration. In turn, triglyceride concentration seems to gradually decrease following kidney transplantation over years [67]. According to some studies, changes in HDL and triglycerides and lipoprotein profile following kidney transplantation depend on the successful engraftment and maintenance of graft function. It has been demonstrated that HDL concentration once again decreased and triglyceride concentration increased if graft function was not maintained [66,67].

## 3. Risk Associated with Bad Cholesterol Profile and Benefits Associated with Lipid Lowering in CKD Patients

The increase in cardiovascular morbidity/mortality in CKD patients is associated with the presence of oxidative stress and inflammation [7,68]. The first condition activates transcription factors which leads to the release of proinflammatory cytokines and the activation of macrophages, while the latter is related to the production of reactive oxygen species (ROS) and the promotion of oxidative stress in tissues [68]. State of increased oxidative stress in CKD patients is believed to be associated with higher concentrations of uremic toxins [68]. Also, the levels of antioxidant enzymes, including superoxide dismutase, catalase, and glutathione peroxidase have been shown to be diminished in CKD patients [68].

In healthy state, HDL cholesterol exert anti-inflammatory and antioxidant properties and it hinders monocyte infiltration in artery intimal walls, thus impeding the development of atherosclerosis [69]. In CKD, impeded HDL cholesterol maturation is associated with diminished apoprotein (Apo) A-1 level. As HDL cholesterol participates in reverse cholesterol transport and it prevents macrophages from accumulating cholesterol and forming foamy cells, the deficiency ApoA-1 can result in both impaired HDL binding to ATP binding cassette transporter A-1 and in free cholesterol efflux from macrophages to HDL cholesterol [70]. Free cholesterol accumulate in macrophages resulting in the formation of foamy cells in vessels and in consequence to the development of atherosclerotic plaques [7]. In CKD patients, the dysfunction of HDL cholesterol is associated with its impaired anti-oxidant properties because of decreased ability to reduce the production of reactive oxygen species (ROS) and monocyte chemoattractant protein-1 which results in lower ability to limit monocyte infiltration and to hinder endothelial adhesion molecule expression [70]. The upregulation of the acyl-CoA cholesterol acyltransferase results in a decrease of both the release of intracellular cholesterol and also the ability of HDL cholesterol to limit the formation of oxidized LDL cholesterol levels as a consequence of paraoxonase and glutathione peroxidase deficiency in CKD patients [69]. The aforementioned alterations are associated with enhanced oxidative stress and elevated cardiovascular mortality in CKD patients [7]. 

Apart from HDL, kidney disease, may also influence the level of LDL. However, the composition of LDL particles is much more affected by the presence of chronic kidney disease than their concentration. In CKD, more atherogenic small and dense LDL particles are more abundant than in general population [71]. Chen et al. [72] studied the advanced CKD-induced changes in the lipidomic profile and demonstrated elevated levels of free fatty acid, glycerolipid, and glycerophospholipid and saturated fatty acids (methyl hexadecanoic acid and 3-oxooctadecanoic acid) in comparison to the control group. Increased free fatty and saturated fatty acid levels were shown to enhance the risk for CVD in CKD patients. Decreased content of phosphadylcholine, plasmenyl ethanolamine, sulfatide, ceramide, and cholesterol sulfate levels in LDL cholesterol structure of CKD patients and increased levels of triacylglyceride and *N*-acyltaurine could stimulate atherosclerotic plaque formation even in the absence of the inflammatory markers and normal levels of oxidized LDL cholesterol [73]. Despite the fact that the levels of serum LDL cholesterol can be within normal range in CKD patients, the concentration of atherogenic sdLDL and the risk of atherosclerotic plaque formation increases along with the progression of kidney function deterioration [16]. Therefore, it seems reasonable to analyze the cholesterol profile in detail in this group of patients [7]. 

Another mechanism related to enhanced cardiovascular CKD risk is the detrimental effects of CKD on the rate of HDL-mediated reverse cholesterol uptake from peripheral tissues and unloading this cholesterol cargo in the liver [34,74]. Defective HDL-mediated reverse cholesterol uptake is associated with the downregulation of lecithin-cholesterol acyltransferase, the decreased production and the enhanced catabolism of apolipoprotein A1 as well as the upregulation of acyl-coenzyme A cholesterol acyltransferase-1 [75]. Moreover, the modifications of apolipoprotein A1 result in the impairment of HDL ability to bind to the machinery that mediates cholesterol efflux via ATP-binding cassette transporter A1 and G1 [34,76]. These apo-AI modifications may also limit the binding of HDL to scavenger receptor-B1, which in consequence leads to the defective disposal of HDL-C cargo in the liver [34,77].

### 3.1. Children/Adolescent Population

According to US Renal Data System: USRDS 2011 [78] rates of cardiovascular death in children on peritoneal dialysis and hemodialysis are similar; however transplant recipients have a relatively decreased risk of cardiac death. Because of the fact that CKD and dialysis are relatively unusual in childhood, predicting the cardiovascular risk in this population is more difficult [79]. However, it seems that reasons for cardiovascular-related mortality are slightly different in children with CKD in comparison to adults with CKD. Adult cardiovascular deaths are primarily associated with coronary artery disease and congestive heart failure [80,81]. Cardiac arrest is the most common cause of CKD children deaths and it is followed by arrhythmia, cardiomyopathy, and cerebrovascular disease. Atherosclerosis is evident in children with advanced kidney disease even though the symptomatic CAD is rarely seen in this population [82]. Järvisalo et al. [83] demonstrated the presence of features of subclinical atherosclerotic cardiovascular disease and an increase in intimal medial thickness of the aorta and carotid arteries in children with familial hypercholesterolemia. The results of numerous studies confirm that atherosclerotic cardiovascular disease begins in childhood, and that dyslipidemia in children in general population may be of key role in its pathogenesis. LDL level has been found to be associated with atherosclerotic disease of the aorta and coronary vessels of children [84]. Another study demonstrated increased TC and decreased albumin levels in children undergoing dialysis who died, which may indicate the role of these disturbances in mortality [85]. Despite the fact that this study, after adjusting for age, failed to demonstrate the significant relationship between TC or albumin and mortality, it seems that the correction of TC and albumin levels in all patients with CKD is important since the results of many studies indicate that these risk factors are predictors of morbidity and mortality in CKD.

### 3.2. Adult Population

Numerous mechanisms play a considerable role in the formation of atherosclerotic plaque related to HDL cholesterol deficiency and dysfunction [7]. The results of studies concerning the association between cholesterol level and cardiovascular risk in patients with chronic kidney disease are conflicting. Some of them have found such relationship [86], some failed to do so [87,88], and some demonstrated the presence of inverse association between cholesterol levels and mortality (reverse epidemiology) [89]. Increased levels of cholesterol are, in general, associated with increased CAD mortality in patients with stage 1–4 CKD. In dialysis patients, a reverse association between serum cholesterol levels and mortality is observed. According to numerous studies, the relationship between ESRD mortality and cholesterol level is in the form of U-shaped curve [90,91]. A large database analysis revealed that patients with total cholesterol levels between 200 and 250 mg/dL had the lowest risk for death, whereas those with levels above 350 mg/dL had a 1.3-fold relative risk and those with levels of 100 mg/dL had a 4.2-fold unadjusted relative risk [92]. It has been suggested that the inverse relationship between cholesterol and ESRD mortality may be related to effects of systemic inflammation and malnutrition, which are prevalent in dialysis patients, not only to their effects on high cholesterol [16,90]. Enhanced systemic inflammation and oxidative stress promote the oxidation of LDL cholesterol [16,69]. In patients with end-stage renal disease the relation between low cholesterol and mortality cannot be explained by significant effects of inflammation and malnutrition as in ESRD patients without these complications the association between dyslipidemia and CVD is still observed [93,94]. It seems that cardiovascular risk may be associated not with levels of cholesterol but the higher occurrence of more atherogenic subfractions which are not measured during standard cholesterol profile test, therefore the results of studies analyzing the relationship between lipoproteins levels and cardiovascular mortality may be conflicting.

The U-shaped curve depicting the association between TC and mortality seemed more linear after the adjustment for serum albumin [93,95,96]. The thesis concerning the impact of inflammation and malnutrition in dialysis patients was confirmed by large, 10-year prospective study of Japanese HD patients [93] which has shown independent association between low TC and higher C-reactive protein (CRP) and mortality in patients with low albumin. In another prospective study of dialyzed patients an increase in baseline TC of 1 mmol/L was associated with a decrease in all-cause mortality in the presence of inflammation/malnutrition [96].

According to studies, the excess risk associated with increased LDL-C diminishes along with the decline in eGFR [97]. Tonelli et al. [98] demonstrated that the hazard ratio [HR] (95% confidence interval [CI]) of incident myocardial infarction (MI) related to LDL-C 44.9 mmol/L (in comparison to 2.6–3.39 mmol/L [100–131 mg/dL]) is 3.01 (2.46–3.69), 2.30 (2.00–2.65) and 2.06 (1.59–2.67) for patients with eGFR of ≥90, 60–89.9 and 15–59.9 mL/min/1.73 m^2^, respectively [97,98]. The relationship between LDL-C and MI risk seems linear at LDL-C above 2.6 mmol/L (100 mg/dL). Experimental studies have confirmed that dyslipidemia boosts lipid peroxidation and triggers free radical reactions [3,4].

Numerous studies have confirmed that the risk of death following MI is elevated in the group of people with CKD in comparison to people with normal kidney function, and it is especially high in those undergoing dialysis [97,98,99,100]. The results of retrospective study of 12,000 HD patients revealed that mortality risk in patients with low TC (<100 mg/dL [2.6 mmol/L]) was over 4 times higher when compared with patients with TC levels between 200 and 250 mg/dL (5.2–6.5 mmol/L) [91]. Kanda et al. [101] suggested that apart from lipoprotein cholesterol levels, also the subclass compositions might be related to enhanced risk of death in CKD population.

Recent study demonstrated stepwise increase in risk of CKD [hazard ratio (HR): 1.05 (95% confidence interval [CI]: 0.97 to 1.13)] and peripheral arterial disease (PAD) [HR: 1.41 (95% CI: 1.23 to 1.62)] with higher LDL-C in individuals with LDL-C above the 95th percentile in comparison to those whose LDL level was below the 50th percentile [102]. Genetic, causal analyses revealed that risk ratio associated with 1 mmol/L higher LDL-C was 3.83 (95% CI: 2.00 to 7.34) for CKD and 2.09 (95% CI: 1.30 to 2.38) for PAD.

According to large randomized Study of Heart and Renal Protection (SHARP) in patients with lower LDL level following treatment (simvastatin 20 mg daily plus ezetimibe 10 mg daily) a significant 17% reduction in the risk of combined major atherosclerotic events, including ischemic stroke, non-fatal myocardial infarction, or coronary revascularization procedures is reported [103,104]. A meta-analysis of 50 trials failed to show the improvement in all-cause mortality in statin-treated CKD patients with significantly reduced lipid concentrations [105].

Moreover, renal lipid deposition has been shown to negatively influence the progression of renal disease itself and therefore a hypothesis was formulated that targeting dyslipidemia in CKD can help to delay the progression of renal disease [106]. Immunohistological analyses of apoB/apoE amount in the glomeruli in renal disease found its association with accelerated progression of the renal disease itself [107]. Also, the results of meta-analysis which included several small, older studies implied that the rate of decline in GFR was reduced in patients receiving a lipid-lowering agent [108]. However, some prospective cohort studies failed to find any relationship between lipid levels and kidney disease progression, thus this association needs further assessment [109]. Also, in the SHARP study no benefits of lipid lowering therapy on the progression of renal disease in patients with 3–5 CKD stages were observed [103].

Some papers demonstrated that statin and other anti-hyperglycemia agents could reduce oxidative stress. For example, Beltowski et al. suggested that statins could diminish the formation of reactive oxygen species by influencing vascular NAD(P)H oxidase and the antagonizing of pro-oxidant effect of angiotensin II and endothelin-1 [110]. Moreover, they can increase the synthesis of vascular nitric oxide, hamper the respiratory burst of phagocytes, or even exert direct free radical scavenging activity [110]. Statin actions involve the inhibition of atherogenesis, stabilization of atherosclerotic plaque, hampering of myocardial hypertrophy, and remodeling, as well as the modulation of vascular tone. Sørensen et al. [111] demonstrated that statin treatment protected from oxidative stress-related DNA and RNA damage. Finally, Rodriguez et al. [112] suggested that statin were also capable of attenuating inflammatory process.

The summary of the results of studies analyzing the relation between lipoproteins levels and cardiovascular mortality/morbidity is presented in Table 1.

## 4. Guidelines and Recommendations Concerning Lipid Levels and Therapeutic Lifestyle Change (Including Diet) in CKD Patients

According to the Kidney Disease: Improving Global Outcomes (KDIGO) guidelines in adults with newly identified CKD, the determination of a lipid profile (TC, LDL, HDL, and triglycerides) should be performed primarily in order to detect the potential severe hypercholesterolemia or hypertriglyceridemia and potential secondary cause establishment [97]. While there is no particular evidence concerning the usefulness of lipid status determination and its potential to improve clinical outcomes, triglyceride levels >11.3 mmol/L [988.8 mg/dL] (or LDL levels >4.9 mmol/L [189.5 mg/dL] may require further assessment. According to KDOQI Clinical Practice Guidelines for Managing Dyslipidemias in Chronic Kidney Disease in adults with stage 5 CKD and LDL ≥100 mg/dL (≥2.59 mmol/L), the target LDL should be reduced to <100 mg/dL (<2.59 mmol/L) (Guideline 4, level of evidence B) [113]. In turn, in adults with stage 5 CKD and LDL <100 mg/dL (<2.59 mmol/L), fasting TG ≥200 mg/dL (≥2.26 mmol/L), and non-HDL cholesterol ≥130 mg/dL (≥3.36 mmol/L), non-HDL cholesterol should be lowered to <130 mg/dL (<3.36 mmol/L) (Guideline 4, level of evidence C). In case of adolescents with stage 5 CKD and LDL ≥130 mg/dL (≥3.36 mmol/L), the target LDL should be less than 130 mg/dL (<3.36 mmol/L) (Guideline 5, strength of evidence C) [113]. If in adolescents with Stage 5 CKD, the level of LDL is <130 mg/dL (<3.36 mmol/L) but fasting triglycerides are ≥200 mg/dL (≥2.26 mmol/L), and non-HDL cholesterol is ≥160 mg/dL (≥4.14 mmol/L), the reduction of non-HDL cholesterol to <160 mg/dL (<4.14 mmol/L) should be considered (Guideline 5, strength of evidence C). According to KDOQI Clinical Practice Guidelines for Managing Dyslipidemias in Chronic Kidney Disease, in adolescents, the isolated hypertriglyceridemia should be treated with therapeutic lifestyle change [113].

The management of dyslipidemia, regardless of the presence of chronic kidney disease, should be initiated by the introduction of therapeutic lifestyle changes [114]. The limitation of excess dietary fat has been demonstrated to lower the total cholesterol and LDL-C and diminish insulin resistance in children without CKD; however, the evidences concerning the improvement of clinical outcomes in the population pediatric CKD patients are not satisfactory [114,115]. Currently valid 2003 Kidney Disease Outcomes Quality Initiative (K/DOQI) Clinical Practice Guidelines for Managing Dyslipidemias in CKD which is based on the Adult Treatment Panel III (ATP III) Guidelines from the National Cholesterol Education Program suggesting that lifestyle changes comprising the reduction of saturated fat to less than 7% of calories and cholesterol to less than 200 mg/day in patients with low density lipoprotein cholesterol (LDL-C) above the goal should be introduced [116,117,118]. In contrast, 2005 K/DOQI Clinical Practice Guidelines for Cardiovascular Disease in Dialysis Patients recommended a caution in using diet in a population of dialysis patients because of the lack of solid evidences and also to the fact that nutrition guidelines are extrapolated from the general population [116,119]. This attitude has not changed in the 2013 Kidney Disease Improving Global Outcomes (KDIGO) guidelines, which put emphasis on pharmacotherapy aiming at managing dyslipidemia but not on dietary interventions [120]. According to 2003 K/DOQI dietary guidelines concerning the management of dyslipidemia in adult patients with CKD, therapeutic life-style changes involving the limitation of dietary cholesterol to <200 mg per day, are recommended for persons with: TG > 500 mg/dL (≥5.65 mmol/L), LDL-C > 100 mg/dL (≥2.59 mmol/L) and TG ≥ 200 mg/dL (≥2.26 mmol/L) and non-HDL-C ≥ 130 mg/dL [117]. In case of patients with fasting triglycerides ≥1000 mg/dL (≥11.29 mmol/L), the ATP III recommends diet which include a very low-fat diet (<15% total calories), medium-chain triglycerides, and fish oils in order to limit the intake of some long-chain triglycerides [113,121].

Yu-Poth et al. [122] suggested that therapeutic lifestyle changes in some patients with LDL 100–129 mg/dL (2.59–3.34 mmol/L) may be sufficient to reach the goal of LDL <100 mg/dL (<2.59 mmol/L). Therefore, it seems rational to introduce dietary changes 2–3 months before beginning drug treatment in patients with LDL 100–129 mg/dL (2.59–3.34 mmol/L). Shoji et al. [50] demonstrated that in hemodialysis patients remnant lipoproteins (VLDL and IDL) were increased, while HDL was lower even in those with normal or near-normal triglycerides. This may imply that the threshold for triglyceride in the therapy of non-HDL cholesterol in hemodialysis patients should be lower. Despite this and because of the fact that data from randomized trials in hemodialysis patients is lacking, the Work Group concluded that higher threshold of triglycerides (as recommended in the ATP-III) should be used [113]. This assumption means that only patients with very high VLDL and IDL will be treated and therefore further studies are required to establish whether therapy targeting lower levels of VLDL and IDL would be safe and effective in patients with CKD. 

Table 2 summarizes the results of studies concerning the effect of lipid disorders on cardiovascular risk and mortality in CKD patients.

## 5. Diets Helping to Lower Cholesterol Level

In general population, but also in some patients with CKD, it has been indicated that low-fat diets together with greater physical activity enhances HDL and decrease triglycerides levels. The introduction of diet change has been shown to exert beneficial effect on lipid level lowering that reaches beyond pharmacological therapy [113]. According to studies, safe and effective amelioration of lipid profile, especially the lowering of remnant lipoproteins levels could help to diminish the incidence of atherosclerotic cardiovascular disease in patients with CKD. Indeed, several cross-sectional studies have reported that hemodialysis patients have higher risk than comparable patients in the general population [123,124].

In general population, the consumption of healthy diet containing high amounts of vegetables, fruits, whole grains, and fish is believed to reduce the risk of cardiovascular morbidity and mortality because of its impact on lipids, glucose, and blood pressure [124,125,126]. However, the effect of this diet on the progression and mortality of CKD patients is controversial perhaps because of the fact that it is difficult to establish what kind of products are “highly beneficial” for this group of patients [124,127,128]. According to some studies, the modifications of diet may be of key importance in the etiology and the progression of CKD because of the fact that it can modify the systemic adverse processes affecting the kidney function (including glomerular injury, macrovascular and microvascular diseases, as well as arterial hypertension) and alter the risks of non-communicable diseases occurrence (e.g., diabetes mellitus) [129]. Dietary components may modify lipid levels, blood pressure, oxidative stress, inflammatory processes, insulin sensitivity, and many others [130,131]. Dyslipidemia in CKD patients may be improved with adequate diet [132]. It seems that diet might influence the risk factors for kidney injury and cardiovascular disease [129]. It remains to discover whether the influence of diet on risks factors for cardiovascular events, such as serum lipids, blood pressure, and oxidant status alters the clinical outcomes also in patients with chronic kidney disease [129]. Dietary and lifestyle interventions may be also used to decrease adverse outcomes in CKD [75,124].

Traditional Mediterranean diet (MD) is based on high amounts of olive oil, unrefined cereals, and cereal products (such as whole grain bread and pasta, brown rice, etc.,), legumes, fruits and vegetables, moderate to high intake of fish and dairy products, moderate consumption of wine and eating low amounts of meat and meat products seems to be beneficial in CKD [3,133]. It is considered to exert antiatherogenic effects to hamper lipoprotein peroxidation and to favor the maintenance of proper endothelial function [3,134,135,136]. When vegetable oils are treated as the main source of lipids and the intake of energy does not exceed the expenditure, there is even no need to restrict the consumption of lipids [137]. A prospective randomized trial study involving patients with a glomerular filtration rate (GFR) of 60–89 mL/min and dyslipidemia (triacylglycerols > 1.7 mmol/L) and/or total cholesterol > 5 mmol/L (193.35 mg/dL)) demonstrated 26% decrease in TG concentration after 90 days of initiating nutritional intervention in the study group in comparison to a control group, while total cholesterol (TC) concentration was reduced by 14% after 60 days and by 35% after 90 days (*p* < 0.05) [3]. Moreover, in case of TC, its level after 90 days was lower than at the baseline (*p* < 0.05). Also, the TC/HDL-C ratio was diminished after 30, 60, 90 days, while apo A-I/apo B ratio was increased after 90 days in the intervention group compared to the control group and to T0 (*p* < 0.05). The consumption of MD diet seems not to considerably influence the concentrations of HDL-C, apo A-I, and apo B [3]. Also in other studies, the beneficial effects of MD diet on hypertriglyceridemia have been observed [138,139,140]. Expert Panel on Detection and Treatment of High Blood Cholesterol in Adults suggested the presence of a negative correlation between monounsaturated fats (MUFA) intake (mainly olive oil) and TC in the intervention group. MUFAs possess antioxidant properties and hamper the release of arachidonic acid from the lipid constituents of cell membranes [134]. Also, Stachowska et al. [141] observed that Mediterranean diet reduced the serum LDL cholesterol levels in comparison to a low fat diet (MD −0.60 mmol/L (−23.20 mg/dL), 95% CI −1.15 to −0.05). Mekki et al. [3] suggested on the basis of the results of their study that the introduction of MD diet in patients with chronic renal failure before dialysis improved food consumption, decreased dyslipidemia, and protected against lipid peroxidation and inflammation. Owing to that patients starting dialysis were in acceptable nutritional and cardiovascular state [3]. The study of 21 patients after kidney transplantation being on Mediterranean diet (MD) and control group consisting 16 patients who also underwent such transplantation, who were consuming low-fat diet revealed a reduction in cholesterol level during the first months on MD diet only in the group of young and middle-aged patients [141]. In turn, slight lowering of cholesterol level was seen among elderly patients. According to authors, Mediterranean diet could be ideal for post-transplantation patients without severe pathologic dyslipidemia [141]. Pharmacologic therapy reducing pro-atherosclerotic lipid levels should be introduced in patients with substantial dyslipidemia in combination with this diet. Ricardo et al. [127] demonstrated that a diet rich in fruits and vegetables and poor in saturated fat and sodium diminished the rates of age-adjusted all-cause mortality in individuals with CKD. Also, the results of other epidemiologic studies have implied that a diet high in fruits and vegetables and low intake of salt and sugar might be beneficial in CKD population [142].

In turn, Salmean et al. [143] demonstrated that high fibers diet (23 grams per day) for six weeks improved lipid profile in CKD patients which resulted in considerable reduced total cholesterol, LDL, and cholesterol-HDL ratio. Resveratrol-dietary supplement present in some kinds of foods and herbal medicines-has been shown to ameliorate lipid handling and mediate anti-atherogenic effects on cholesterol flux in murine and cell culture models [144]. It has been suggested that resveratrol improved oxidant status and inhibited lipid peroxidation in hypertensive rat model [145]. At the same time it proved to be safe, well-tolerated, inexpensive and it could be used in combination with other therapies [146]. The intake of isolated fibers, specifically viscous fibers, but also of whole food containing high amount of fibers has been associated with the diminished blood glucose response and reduced LDL and total cholesterol [147,148,149]. Salmean et al. [143] suggested that the addition of fiber into commercially available foods consuming by individuals with CKD could result in the improvement of their quality of life, without impacting the clinical markers and symptoms. In their small study (15 patients with stage 3 to 5 CKD) in which participants were provided with control foods containing <2 g/day of fiber for 2 weeks (control period) or control foods containing <2 g/day of fiber for 2 weeks, followed by similar foods providing 23 g/day for 4 weeks (fiber intervention period) as an addition to their usual diet, the decrease in total cholesterol from 175 ± 12 mg/dL (4.53 ± 0.31 mmol/L) to 167 ± 11 mg/dL (4.32 ± 0.28 mmol/L) (*p* = 0.02) was observed. Moreover, a strong trend was demonstrated for decreased LDL cholesterol (100 ± 8 mg/dL (2.59 ± 0.21) to 93 ± 7 (2.40 ± 0.18); *p* = 0.05) and a decline in TC:HDL ratio from 4.0 ± 0.3 during control to 3.7 ± 0.2 during the fiber intervention (*p* = 0.02) period. The addition of fiber into diet did not alter considerably the level of HDL cholesterol and TG [143]. Krishnamurthy et al. [150] proposed that higher intake of fiber was related to reduced inflammation and mortality in CKD.

Fontes et al. [132] found that a low-protein diet prescribed for six months to patients with pre-dialysis CKD not only reduced total cholesterol and LDL-C but also helped to preserve renal function and diminished serum levels of uric acid. According to authors the lowering of serum levels of total cholesterol and LDL-C observed in this study was not surprising. The limitation of animal protein consumption did not alter the percentage of lipid intake; however, it was associated with a noteworthy reduction in the intake of dietary cholesterol. This finding supports the view that low-protein diets results in the reduction in dietary cholesterol intake. Some recent data imply that dietary cholesterol, apart from heightening LDL-C, also increases HDL-C concentration and the level of less atherogenic large buoyant LDL and it may improve HDL functional properties, and to a small extent, it can impact the ratio of LDL-C/HDL-C [116].

Study by Lai et al. [151] including patients with CKD stages 3 and 4, consuming low-protein diet for 12 months found only insignificant improvements in lipid profiles: the reduction in TC, LDL-C, and triglyceride levels, and increase in HDL-C level. Some reviews suggested that the reduction of fat consumption and increased intake of fruit, vegetables, and fiber can diminish the serum cholesterol and lower arterial blood pressure by up to 10 mm Hg [129,152,153]. The purpose of the introduction of diet comprising ketoanalogs of amino acids (KAs) is to reduce renal death, delay the progression of CKD, avoid hyperphosphatemia and hyperparathyroidism, and improve blood pressure control in patient with CKD [154]. A meta-analysis of effects of diet based on ketoanalogs of amino acids (KAs) supplements on cholesterol levels in CKD patients failed to demonstrate significant reduction of cholesterol level (MD = −24.13, 95% CI = (−93.68, 45.42), *p* = 0.50) [154]. The results of this analysis suggest that a very-low-protein diet also does not significantly lower the serum cholesterol level.

Studies on animal models indicate that a vegetarian diet is suitable and nutritionally adequate in chronic kidney disease [155]. A vegetarian diet brings cardiovascular benefits as it lowers blood pressure, improves glycemic control in diabetic and insulin-resistant individuals, and ameliorates lipid profile [156]. Mukkuden-Petersen et al. [157] demonstrated the decrease in TC and LDL cholesterol in vegetarians in comparison to persons on a meat diet. The improvement of lipid profile in vegetarians is related to the high content of unsaturated fatty acids present in nuts, soy, and plant sterols [158]. The effect of vegetarian diet were observed in a large Finnish study which revealed inverse relationship between intake of vegetables and the risk of coronary artery disease and cardiovascular deaths [159]. Also the meta-analysis of results of five prospective studies comprising over 750,000 patients showed 24% lower mortality from ischemic heart disease in vegetarians compared to non-vegetarians (death rate ratio: 0.76; 95% CI: 0.62, 0.94; *p* < 0.01 after over 10 years of follow-up [160]) without affecting TC or Ca levels. The analysis of the impact of vegetarian diet on patients undergoing hemodiafiltration (HDF) demonstrated lower levels of indoxyl sulfate (IS) and p cresyl sulfate (PCS) (toxins which accumulate in CKD) [158]. According to numerous studies, vegetarian diet supplemented with a very-low-protein diet is safe for pre-dialysis and dialysis patients, as it exerts no detrimental effect on their short- and long-term outcomes [161]. However, patients with problems with potassium levels should avoid potassium-rich plant proteins. A well-planned vegetarian diet seems to be related with cardiovascular benefits as well as the correction of CKD-accompanying complications.

Some studies analyzed the effect of vitamin supplementation on cardiovascular risk in CKD patients [7]. Antioxidant effects of vitamin E has been shown to decrease CVDs in hemodialysis patients in randomized, placebo-controlled trials [162]. Also, Islam et al. [163] demonstrated that alpha-tocopherol diminished the LDL susceptibility to oxidation and therefore it exerted protective effect against cardiovascular complications in CKD patients undergoing dialysis therapy. Moreover, vitamin E supplementation was found to improve lipid profile in hemodialysis patients [164]. A recent systematic review and meta-analysis showed that antioxidants may delay diabetic kidney disease progression and help to ameliorate kidney function following early renal damage [165].

The treatment of dyslipidemia is of key importance in the management of CKD and diabetic kidney disease (DKD) in order to reduce elevated risk of cardiovascular disease and death; however, until now the optimal dietary fat intake has not been established [166,167,168]. Therapeutic lifestyle changes involving dietary fat restriction has been shown to be safe also in children since it has no effect on proper growth and development, or nutrition [169,170]. Orozco et al. proposed that the combination of appropriate diet and exercise have modest effects on blood lipids and blood pressure in people at risk of diabetes, many of whom suffered from kidney disease [171]. Because of the fact that the consumption of saturated fatty acids (SFA) and trans-fat increases the risk of cardiovascular disease, they should be avoided, while Omega-3 and 6 polyunsaturated fatty acids (PUFAs) and monounsaturated fatty acids (MUFAs) are considered as beneficial since they limit inflammation and endothelial dysfunction and improve dyslipidemia [166,172]. A nested case-control, prospective cohort of low-income blacks and whites in the south-eastern United States demonstrated a marginally significant inverse trend between higher dietary polyunsaturated fatty acids (PUFA) consumption and lower incidence of ESRD [173]. These findings are in agreement with the results of other studies indication protective effect of omega-3 upon cardiovascular outcomes [172,174]. A diet that comprises one to two servings of oily fish, such as salmon, mackerel, herring, sardines, bluefish, and anchovies per week can lower triglyceride levels and diminish the risk of death from coronary heart disease because of high content of docosahexaenoic acid (DHA) and eicosapentaenoic acid (EPA) [175]. However, it remains unraveled whether fish oil supplements can exert the same beneficial effects. Some studies demonstrated that higher doses of supplements can improve the cardiovascular outcomes. Some studies also assessed in patients with CKD the effects of fish oil supplements on lipoproteins, which was found to decrease triglycerides in the general population, however the obtained results were questionable [113,176,177].

Also, a diet comprising high amount of soy protein and precisely isoflavones can to some extent reduce levels of total cholesterol, LDL cholesterol, and triglycerides and increase levels of high-density lipoprotein (HDL) cholesterol. Also soy foods and food products (e.g., tofu, edamame, and soy butter) may exert beneficial effects on lipids and cardiovascular health because of the fact that they are deficient in saturated fats and abundant in unsaturated fats. Another natural diet compounds which block the absorption of cholesterol in the intestine thus being beneficial are plant stanols and sterols, naturally found in some fruits, vegetables, vegetable oils, nuts, seeds, and legumes [175]. Tallman et al. [116] demonstrated favorable effect of bioactive components of eggs on lipoprotein particle profiles and HDL functionality in heathy adults. However, it remains unknown whether their impact on lipid parameters ESRD population would be the same.

Currently, there are no randomized trials which have assessed the safety and efficacy of a low-fat, low-cholesterol diet in patients with CKD. However, the results of studies comprising persons from the general population imply that a lipid-lowering diet can reduce LDL [121,122,178]. Such diet should comprise <7% of calories in the form of saturated fat, not more than 10% of calories in the form of polyunsaturated fat, less than 20% of calories as monounsaturated fat, total fat amount not exceeding 25% to 35% of total calories, complex carbohydrates (50% to 60% of total calories), and fiber (20–30 g per day) [113]. 

Further studies are required to assess in detail the dietary components which consumption improves health of individuals with CKD. For sure, in pre-dialysis and dialysis protein-energy malnutrition should be avoided as it is, in population of CKD patients, a strong predictor of adverse outcomes. Moreover, some dietetic limits present especially on ESRD patients concerning liquid and ions (especially potassium) can influence the dietary habits of these patients. Table 3 summarizes the effects of various diets in CKD population.

## 6. Conclusions

Currently available evidence gives the impression that data on dietary interventions in CKD patients is not sufficient to make any clinical practice guidelines and is of low quality [129]. It seems that diet modification may exert beneficial effect on CKD patients’ quality of life, their outcomes, and the progression on kidney disease through the impact on cholesterol levels, blood pressure, and serum albumins. The preliminary results of studies of the influence of diet on CKD patients suggest potential mechanisms for benefits of diet modifications; however they have to be confirmed in larger and longer term studies [129]. However, in the opinion of Nelms et al. [179] in patients with advanced stages of CKD there may be a need to limit foods naturally rich in fiber including whole grains, legumes, and certain fruits and vegetables because of their phosphorous and/or potassium contents. The assessment of impact of diet on any patients’ outcomes is difficult because of the possibility of lack of patients’ compliance with the prescribed diet. Also the facts that various diets were used in wide range of clinical settings in the studies and there are hardly any strong evidences concerning clinical outcomes, no specific dietary guidelines or recommendations can be made for people with CKD, those treated with dialysis or a kidney transplant recipients.

## Figures and Tables

**Table 1:** The summary of results of studies concerning lipid disorders in chronic kidney disease (CKD).

Group of Patients	Type of Study	Finding	Ref.
Hemodialysis patients	Cohort study (*n* = 1167)	Independent association between low TC and higher CRP and mortality in patients with serum albumin values ≥4.5 g/dL (adjusted hazards ratio was 1.370 (1.109 to 1.692), *p* = 0.0034) Lowest mortality rate (35.9%) at the baseline serum cholesterol level of 200 to 219 mg/dL and the highest (57.2%) at the serum cholesterol of less than 140 mg/dL (adjusted hazards ratio 0.939 (0.891 to 0.989), *p* = 0.0180)	[93]
Dialyzed patients	Prospective study	Increase in baseline TC by 1 mmol/L was associated with a decrease in all-cause mortality in the presence of inflammation/malnutrition.	[96]
Adults from the Alberta Kidney Disease Network (excluding V CKD)	Large study (*n* = 836,060)	Relationship between LDL-C and MI risk seems linear at LDL-C above 2.6 mmol/L (100 mg/dL). Adjusted HRs (95% CI) of MI associated with LDL-C of ≥4.9 compared with 2.6–3.39 mmol/L in participants with eGFR = 15–59.9 and 60–89.9 ml/min per 1.73 m^2^ were 2.06 (1.59, 2.67) and 2.30 (2.00, 2.65), respectively	[98]
Hemodialysis patients	(*n* > 12,000)	>4 times higher mortality risk in patients with low TC (<100 mg/dL [2.6 mmol/L]) versus patients with TC levels between 200 and 250 mg/dL (5.2–6.5 mmol/L)	[91]
CKD population (stage 4 and 5)	Prospective cohort study (*n* = 71)	Subclass composition of lipoproteins might be related to enhanced risk of death in CKD population. Cholesterol proportions in very small HDLs were associated with eGFR change rate [F19 β = −17.63, *p* = 0.036] and ankle-brachial index (ABI) (a marker of atherosclerosis in the peripheral artery) [F19 β = 0.047, *p* = 0.047] in stage 4 group.	[101]
CKD patients (3023 on dialysis and 6247 not) with no known history of MI or coronary revascularization	Randomized double-blind trial (*n* = 9270)	Lower LDL level (after statin treatment) was associated with a significant 17% reduction in the risk of combined major atherosclerotic events (526 [11.3%] simvastatin plus ezetimibe vs. 619 [13.4%] placebo; rate ratio [RR] 0.83, 95% CI 0.74–0.94; log-rank *p* = 0.0021) Non-significant association between LDL lowering with non-fatal MI or death from CHD (213 [4.6%] vs. 230 [5.0%]; RR 0.92, 95% CI 0.76–1.11; *p* = 0.37) and significant reduction in non-hemorrhagic stroke (131 [2.8%] vs. 174 [3.8%]; RR 0.75, 95% CI 0.60–0.94; *p* = 0.01) and arterial revascularization procedures (284 [6.1%] vs. 352 [7.6%]; RR 0.79, 95% CI 0.68–0.93; *p* = 0.0036).	[103]
CKD, patients on maintenance dialysis or after renal transplantation	Meta-analysis of randomized and quasi-randomized controlled trials (*n* = 30,144)	No improvement in all-cause mortality in statin-treated CKD patients with significantly reduced lipid concentrations (44 studies, 23 665 patients; 0.92, 0.82 to 1.03). Significant effect of lipid lowering on the occurrence of fatal cardiovascular events (43 studies, 23 266 patients; relative risk 0.81, 0.73 to 0.90) and non-fatal cardiovascular events (8 studies, 22 863 patients; 0.78, 0.73 to 0.84).	[105]
	A meta-analysis of 13 prospective controlled trials	Lower rate of decline in glomerular filtration rate in patients receiving a lipid-lowering agent compared with controls (treated controls, 0.156 mL/min/month; 95% CI, 0.026 to 0.285 mL/min/month, *p* = 0.008).	[108]

**Table 2:** Summary of guidelines concerning lipid disorders in CKD.

Group of Patients	Type of Recommendation	Recommendation	Level of Evidence	Ref.
Adults with newly identified CKD	Kidney Disease: Improving Global Outcomes (KDIGO)	Determination of a lipid profile (TC, LDL, HDL, and triglycerides) should be performed primarily in order to detect potential severe hypercholesterolemia or hypertriglyceridemia and potential secondary cause establishment.		[97]
Adults with newly identified CKD	Kidney Disease: Improving Global Outcomes (KDIGO)	Triglyceride levels >11.3 mmol/L or LDL levels >4.9 mmol/L require further assessment		[97]
Adults with stage 5 CKD and LDL ≥100 mg/dL (≥2.59 mmol/L)	KDOQI Clinical Practice Guidelines for Managing Dyslipidemias in Chronic Kidney Disease. Guideline 4	Target LDL should be reduced to <100 mg/dL (<2.59 mmol/L).	B	[113]
Adults with stage 5 CKD and LDL <100 mg/dL (<2.59 mmol/L), fasting TG ≥200 mg/dL (≥2.26 mmol/L), and non-HDL cholesterol ≥130 mg/dL (≥3.36 mmol/L)	KDOQI Clinical Practice Guidelines for Managing Dyslipidemias in Chronic Kidney Disease. Guideline 4	Non-HDL cholesterol should be lowered to <130 mg/dL (<3.36 mmol/L).	C	[113]
Adolescents with stage 5 CKD and LDL ≥130 mg/dL (≥3.36 mmol/L)	KDOQI Clinical Practice Guidelines for Managing Dyslipidemias in Chronic Kidney Disease. Guideline 5	Target LDL should be less than 130 mg/dL (<3.36 mmol/L).	C	[113]
Adolescents with Stage 5 CKD, LDL <130 mg/dL (<3.36 mmol/L), fasting triglycerides ≥200 mg/dL (≥2.26 mmol/L), and non-HDL cholesterol ≥160 mg/dL (≥4.14 mmol/L)	KDOQI Clinical Practice Guidelines for Managing Dyslipidemias in Chronic Kidney Disease. Guideline 5	Reduction of non-HDL cholesterol to <160 mg/dL (<4.14 mmol/L) should be considered.	C	[113]
Adolescents with CKD	KDOQI Clinical Practice Guidelines for Managing Dyslipidemias in Chronic Kidney Disease.	Isolated hypertriglyceridemia should be treated with therapeutic lifestyle change.		[113]
Patients with LDL-C above the goal limit	2003 Kidney Disease Outcomes Quality Initiative (K/DOQI) Clinical Practice Guidelines for Managing Dyslipidemias	Lifestyle changes comprising the reduction of saturated fat to less than 7% of calories and cholesterol to less than 200 mg/day should be introduced.		[116,117,118]
Dialysis Patients	2005 K/DOQI Clinical Practice Guidelines for Cardiovascular Disease in Dialysis Patients	Caution in using diet due to lack of solid evidences.		[116,119]
Adult patients with CKD and: TG > 500 mg/dL (≥5.65 mmol/L), LDL-C > 100 mg/dL (≥2.59 mmol/L) and TG ≥ 200 mg/dL (≥2.26 mmol/L) and non-HDL-C ≥ 130 mg/dL	2003 K/DOQI dietary guidelines concerning the management of dyslipidemia in adult patients with CKD	Therapeutic life-style changes involving the limitation of dietary cholesterol to <200 mg per day are recommended.		[117]
Patients with fasting triglycerides ≥1000 mg/dL (≥11.29 mmol/L)	ATP III	Diet which include a very low-fat diet (<15% total calories), medium-chain triglycerides, and fish oils in order to limit the intake of some long-chain triglycerides is recommended.		[113,121]

**Table 3:** Summary of diet effects in CKD patients.

Group of Patients	Type of Study	Diet	Effect	Ref.
Patients with GFR of 60–89 mL/min and dyslipidemia (triacylglycerols > 1.7 mmol/L) and/or (TC > 5 mmol/L)	Prospective randomized trial	Mediterranean diet	➢ 26% ↓ TG concentration after 90 days after initiating nutritional intervention in comparison to a control group, ➢ ↓ TC concentration by 14% after 60 days and by 35% after 90 days (*p* < 0.05) ➢ ↓ TC/HDL-C ratio after 30, 60, 90 days (*p* < 0.05) ➢ ↑ apo A-I/apo B ratio after 90 days compared to the control group and to T0 (*p* < 0.05).	[3]
Patients with chronic renal failure before dialysis	Prospective randomized trial	Mediterranean diet	Improved food consumption, ↓ dyslipidemia and protection against lipid peroxidation and inflammation	[3]
Patients after kidney transplantation	Case/control study	Mediterranean diet/low fat diet	➢ ↓ cholesterol level during the first months on MD diet only in the group of young and middle-aged patients. ➢ Diet ideal for post-transplantation patients without severe pathologic dyslipidemia ➢ ↓ Serum LDL cholesterol levels in comparison to a low fat diet (MD −0.60 mmol/L, 95% CI −1.15 to −0.05).	[142]
CKD patients	Case/control study	Diet rich in fruits and vegetables and poor in saturated fat and sodium	➢ The risk of all-cause death in individuals in the second, third, and fourth quartiles of the weighted healthy lifestyle score compared to those in the lowest quartile (adjusted hazard ratio of all-cause mortality: 0.53 (95% confidence interval [CI], 0.41–0.68), 0.52 (95% CI, 0.42–0.63), and 0.47 (95% CI, 0.38–0.60) was not different between these groups. ➢ ↓ Rates of age-adjusted all-cause mortality ➢ No significant association between diet and all-cause mortality after multivariable adjustment	[127]
Stage 3 to 5 CKD patients	Case/control study	High fibers diet (23 grams per day) for 6 weeks	➢ Improved lipid profile, considerable ↓ TC, LDL and cholesterol-HDL ratio. ➢ Improved quality of life ➢ ↓ TC from 175 ± 12 to 167 ± 11 mg/dL (*p* = 0.02) ➢ Strong trend for ↓ LDL (100 ± 8 to 93 ± 7 mg/dL; *p* = 0.05) ➢ ↓ TC: HDL ratio from 4.0 ± 0.3 during control to 3.7 ± 0.2 during the fiber intervention (*p* = 0.02) ➢ No significant effect on HDL and TG	[143]
Patients with pre-dialysis CKD	Case/control study	Low-protein diet (0.6 g/kg/d) for six months	➢ ↓ TC (from 199.7 ± 57.1 to 176.0 ± 43.6 mg/dL, *p* = 0.0001) and LDL-C (from 116.2 ± 48.1 to 97.4 ± 39.1 mg/dL, *p* = 0.001) ➢ Preserved renal function and ↓ serum levels of uric acid (from 6.8 ± 1.4 to 6.2 ± 1.3 mg/dL, *p* = 0.004) ➢ GFR increased from 26.2 ± 9.5 to 28.9 ± 12.7 mL/min (*p* = 0.02).	[132]
Patients with CKD stages 3 and 4	Interventional, single-center study	Low-protein diet for 12 months	Insignificant improvements in lipid profiles - ↓ TC (baseline 176.3 ± 37.5; 12 months 159.3 ± 26.2), and TG levels (baseline 126.6 ± 43.8; 12 months 100.2 ± 36.7), and ↑ HDL-C levels (baseline 47.2 ± 18.5; 12 months 52.9 ± 26.6).	[151]
CKD patients	A meta-analysis	Ketoanalogs of amino acids (KAs) supplements	No significant ↓ TC level (MD = −24.13, 95% CI = (−93.68, 45.42), *p* = 0.50)	[154]
